# The evaluation of immunotherapy and chemotherapy treatment on melanoma: a network meta-analysis

**DOI:** 10.18632/oncotarget.13277

**Published:** 2016-11-10

**Authors:** BaSang CiRen, Xinhua Wang, Ziwen Long

**Affiliations:** ^1^ Department of Medicine, Shigatse People's Hospital, Shigatse, Tibet, 85700, China; ^2^ Department of Dermatology, Shigatse People's Hospital, Shigatse, Tibet, 85700, China; ^3^ Department of Gastric Cancer and Soft-Tissue Sarcoma Sugery, Fudan University Shanghai Cancer Center; Department of Oncology, Shanghai Medical College, Fudan University, Shanghai, 200032, China

**Keywords:** melanoma, chemotherapy, immunotherapy, network meta-analysis

## Abstract

**Background:**

Melanoma is a highly malignant tumor that develops from a neural crest derivative called melanocytes. Chemotherapy is recommended for patients with stage III/IV melanoma. Immunomodulation has also been shown to effectively improve the survival rate of such patients. In the current study, we aimed to perform a network meta-analysis on the therapeutic value of chemotherapy and immunotherapy on melanoma.

**Results:**

Twenty randomized controlled trials (RCTs) were enrolled in the study. Our Results indicated that ipilimumab + nivolumab had the highest response rate among all therapies, pembrolizumab also had a good efficacy with an excellent tolerance. Chemotherapy had a low response rate, high adverse effects and progressive diseases qualities, therefore it is not recommended as a preferred treatment for patients with advanced melanoma.

**Methods:**

The Cochrane library, PubMed and Embase databases were searched for relevant articles. Results of the pair-wise meta-analysis were illustrated by odd ratios (ORs) and corresponding 95% confidence intervals (CIs). Network meta-analysis was performed using a random-effects model under Bayesian framework. Results were illustrated by cumulative ORs and corresponding 95% credible interval (CrIs). The probabilities and outcomes of each treatment were ranked and summarized using the surface under the cumulative ranking curve (SUCRA).

**Conclusions:**

We recommend pembrolizumab as the preferred treatment due to its high efficacy and low adverse effects, combination of ipilimumab and nivolumab could be used in severe symptoms.

## INTRODUCTION

Melanoma develops from melanocytes typically in the skin, but has also been reported to occur on mucosal surfaces where neural crest cells migrate, such as the mouth, pleura and iris [1, 2]. Melanoma is the most common cancer in young adults aged 25 to 29 [3], with an age-standardized incidence rate of 10.2% for males and 9.8% for females in developed countries [4], and 73,780 new cases of melanoma and 9,940 melanoma-related deaths in the United States [5].

Both environmental and genetic conditions are considered as risk factors. Family history, skin type, density of freckles, skin color, eye and hair color, pre- malignant and skin cancer lesions, and actinic damage indicators are all significantly related to melanoma susceptibility [6]. Gene mutations combined with environmental factors, particularly the exposure to UV light, may contribute to the onset of melanoma. Oncogenes including BRAF and the microphthalmia-associated transcription factor (MITF) pathway play an important role in the pathogenesis of melanoma [6].

Surgery is recommended for patients with dissectible lesions and oligometastatic melanoma, whereas, chemotherapy and radiotherapy are recommended for patients with non-dissectible melanoma, and particularly for those already in stage III or IV [7]. Besides these treatments, immunomodulation has also been observed to be effective in improving the survival rate of patients with stage IV melanoma [8].

Sentinel node biopsy is the current procedure used by the American Joint Committee on Cancer (AJCC) and Union for International Cancer Control (UICC) to treat and categorize patients with melanoma [9]. Results of the sentinel node biopsy are a strong prognostic factor in melanoma treatment [10]. Tumor thickness in millimeters (Breslow's depth), depth related skin structures (Clark level), ulceration, lymphatic/perineural invasion, tumor-infiltrating lymphocytes, location of lesion, satellite lesions, regional or distant metastasis, and the type of melanoma are all factors that may influence the prognosis of melanoma [7]. The age-standardized mortality rate of melanoma is about 2% [4]. However, for advanced melanoma, the five-year survival rate could be as low as 10% [8].

Several trials concerning the effect of immunomodulation and chemotherapy on melanoma have been performed. The therapeutic value of chemotherapy, cytotoxic T-lymphocyte associated protein 4 (CTLA4) and programmed death1 (PD-1) antibodies has also been assessed in various other studies [8, 11, 12]. However, we failed to find a robust study that compares their effect on melanoma and thus clinical practice on treatment selection is required for patients with advanced melanoma. In current study, we aimed to perform a network meta-analysis concerning the therapeutic value of chemotherapy and immunotherapy on melanoma.

## RESULTS

### Study characteristics

**Figure 1:**
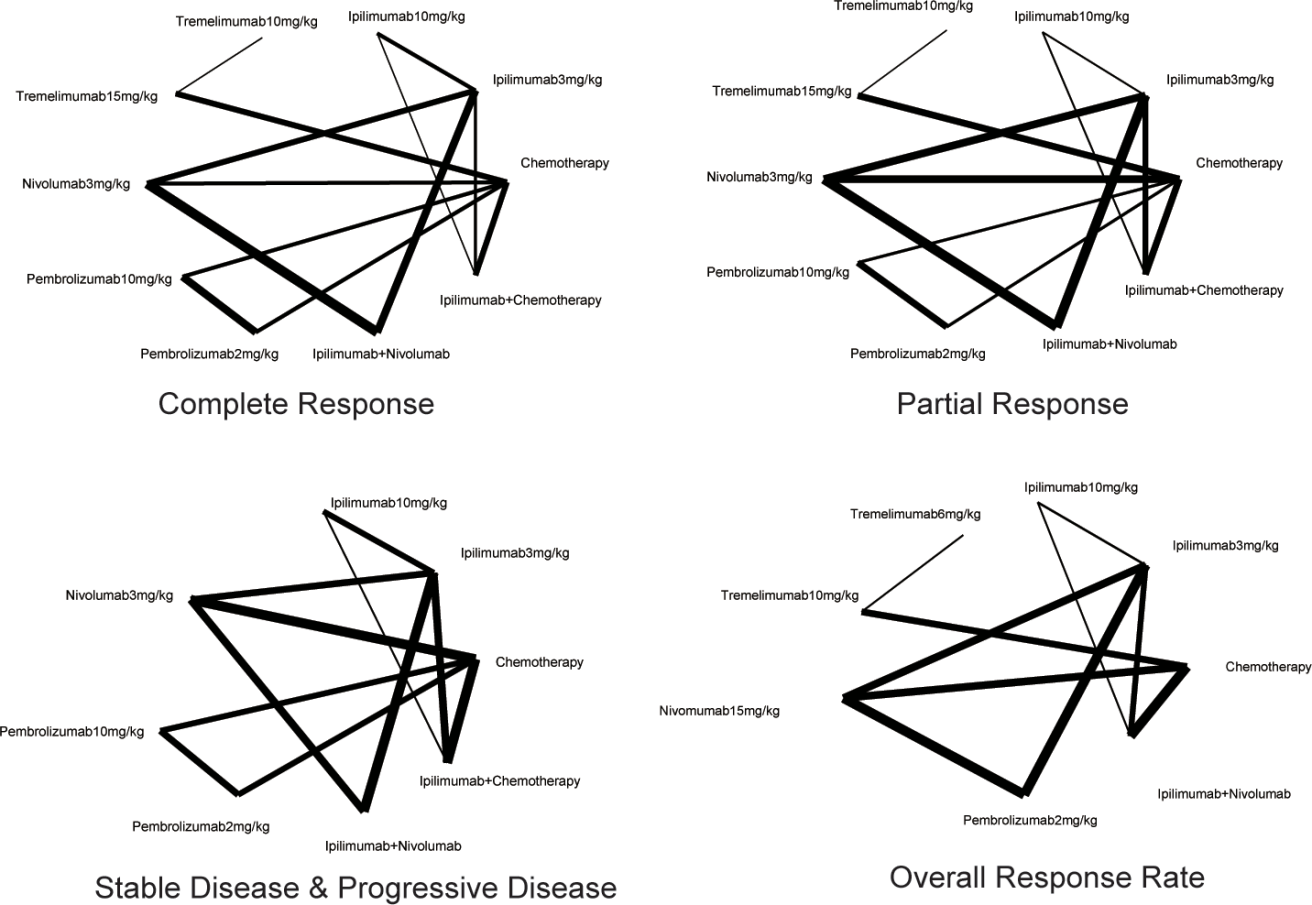
Evidence network of eligible comparisons for complete response, partial response, stable & progressive disease and overall response rate in network meta-analysis The width of the lines represents the cumulative number of trials for each comparison and the size of every node is proportional to the number of enrolled participants (sample size).

**Figure 2:**
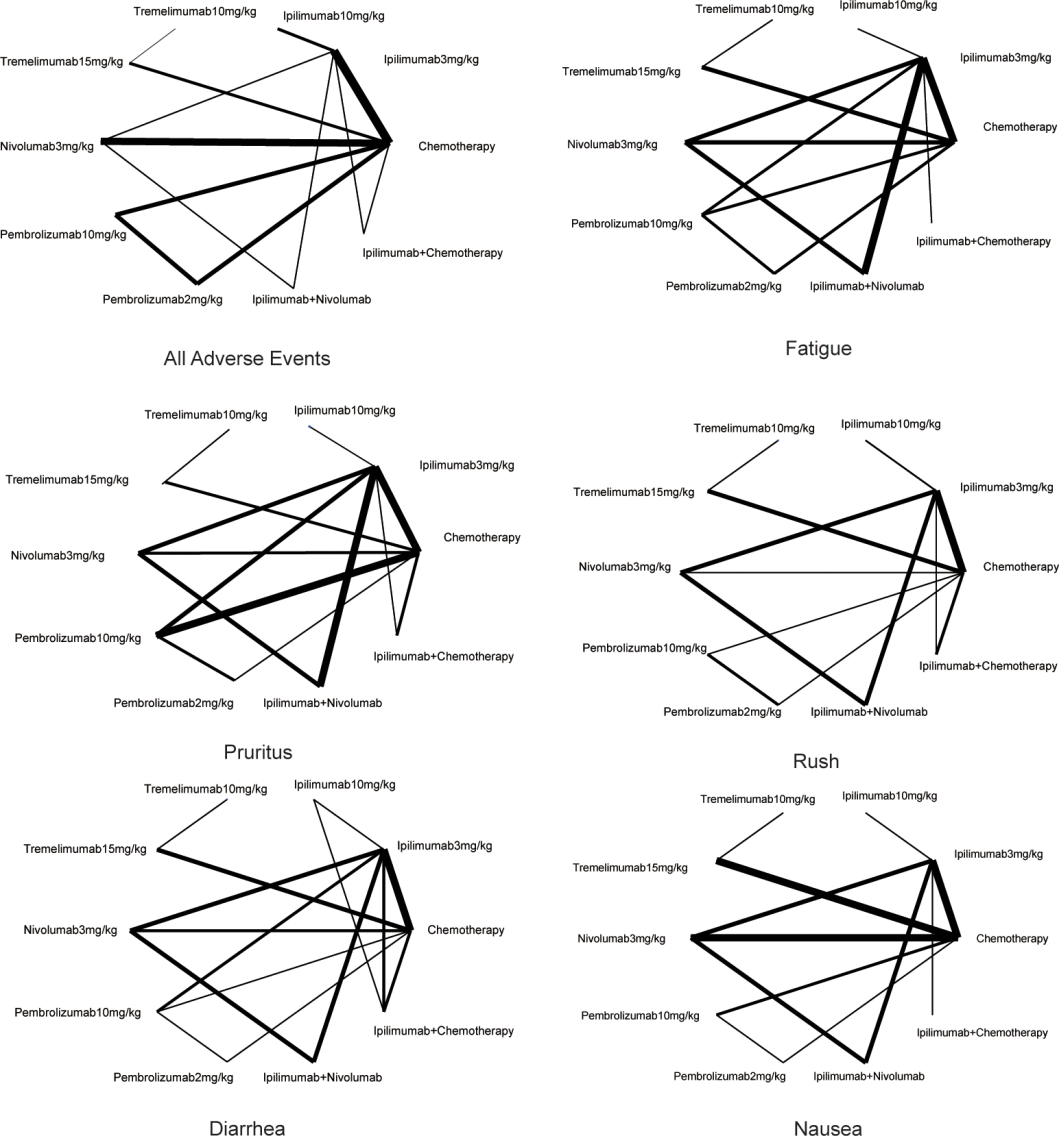
Evidence network of eligible comparisons for various adverse events in network meta-analysis The width of the lines represents the cumulative number of trials for each comparison and the size of every node is proportional to the number of enrolled participants (sample size).

As shown in Table 1 and Supplementary Figure S1, in the present meta-analysis we conducted 20 RCTs concerning the effect of immunotherapy and chemotherapy on patients with melanoma [13–32]. A total of 6,442 cases were involved and interventions were categorized into chemotherapy, ipilimumab 3 mg/kg, ipilimumab 10 mg/kg, tremelimumab 10 mg/kg, tremelimumab 15 mg/kg, nivolumab 3 mg/ kg, pembrolizumab 10 mg/kg, pembrolizumab 2 mg/kg, ipilimumab+nivolumab and ipilimumab+chemotherapy. An evidence network of eligible comparisons regarding outcomes mentioned above all was plotted in Figure 1 and Figure 2.

**Table 1:** Summary of study design characteristics

Study	Trial ID	Trial Phase	Case	Intervention	Outcome
Weber, 2015	NCT01721746	III	405	Nibolumab 3 mg/kg vs. Chemotherapy	CR; PR; SD; PD; ORR; AAE; Fatigue; Pruritus; Diarrhea; Nausea
Robert, 2015	NCT01866319	III	834	Pembrolizumab 10 mg/kg vs. Ipilimumab 3 mg/kg	Fatigue; Pruritus; Rash; Diarrhea
Robert, 2015	NCT01721772	III	418	Nivolumab 10 mg/kg vs. Chemotherapy	CR; PR; SD; PD; ORR; AAE; Fatigue; Pruritus; Rash; Diarrhea; Nausea
Ribas, 2015	NCT01704287	II	540	Pembrolizumab 2 mg/kg vs. Pembrolizumab 10 mg/kg vs. Chemotherapy	CR; PR; SD; PD; AAE; Fatigue; Pruritus; Rash; Diarrhea; Nausea
Postow, 2015	NCT01927419	III/IV	109	Ipilimumab 3 mg/kg+Nivolumab 1 mg/kg vs. Ipilimumab 3 mg/kg	CR; PR; SD; PD; ORR; AAE; Fatigue; Pruritus; Rash; Diarrhea; Nausea
Larkin, 2015	NCT01844505	III	945	Nivolumab 3 mg/kg vs. Ipilimumab 3 mg/kg+Nivolumab 1 mg/kg vs. Ipilimumab 3 mg/kg	CR; PR; SD; PD; ORR; AAE; Fatigue; Pruritus; Rash; Diarrhea; Nausea
Eggmont, 2015	NCT00636168	III	951	Ipilimumab 10 mg/kg vs. Chemotherapy	AAE; Fatigue; Pruritus; Rash; Diarrhea; Nausea
Topalian, 2014	NCT00730639	-	37	Nivolumab 3 mg/kg vs. Nivolumab 10 mg/kg	SD; ORR; AAE; Fatigue; Pruritus; Rash; Diarrhea; Nausea
Robert, 2014	NCT01295827	I	173	Pembrolizumab 2 mg/kg vs. Pembrolizumab 10 mg/kg	CR; PR; SD; PD; ORR; Fatigue; Rash; Diarrhea;
Hodi, 2014	NCT01134614	III/IV	245	Ipilimumab 10 mg/kg+Chemotherapy vs. Ipilimumab 10 mg/kg	CR; PR; SD; PD; ORR; Fatigue; Pruritus; Rash; Diarrhea; Nausea
Ribas, 2013	NCT00257205	III	655	Tremelimumab 15 mg/kg vs. Chemotherapy	CR; PR; ORR; AAE; Fatigue; Pruritus; Rash; Diarrhea; Nausea
Millward, 2013	-	IV	15	Tremelimumab 6 mg/kg vs. Tremelimumab 10 mg/kg vs. Tremelimumab 15 mg/kg	AAE; Fatigue; Pruritus; Rash; Diarrhea; Nausea
Robert, 2011	NCT00324155	III/IV	502	Ipilimumab 10 mg/kg+Chemotherapy vs. Chemotherapy	CR; PR; SD; PD; ORR; AAE; Pruritus; Rash; Diarrhea
Hersh, 2011	NCT00050102	II	76	Ipilimumab 3 mg/kg vs. Ipilimumab+Chemotherapy	PD; ORR; AAE; Fatigue; Pruritus; Rash; Diarrhea; Nausea
Hamid, 2011	NCT00261365	II	82	Ipilimumab 3 mg/kg vs. Ipilimumab 10 mg/kg	CR; PR; SD; ORR
Wolchok, 2011	NCT00289640	III/IV	145	Ipilimumab 10 mg/kg vs. Ipilimumab 3 mg/kg	CR; PR; SD; PD; ORR; AAE; Fatigue; Pruritus; Rash; Diarrhea; Nausea
Weber, 2009	-	III/IV	115	Ipilimumab 10 mg/kg+Chemotherapy vs. Ipilimumab 10 mg/kg	CR; PR; SD; PD; ORR; Diarrhea
Camacho, 2009	NCT0086489	III	115	Tremelimumab 6 mg/kg vs. Tremelimumab 10 mg/kg vs. Tremelimumab 15 mg/kg	CR; PR; AAE; Fatigue; Pruritus; Rash; Diarrhea; Nausea
Ribas, 2005	-	I	20	Ipilimumab 3 mg/kg vs. Ipilimumab 10 mg/kg	Fatigue; Diarrhea; Nausea

CR complete response; PR partial response; SD stable disease; PD progressive disease; ORR overall response rate; AAE all adverse events.

### Clinical outcomes

In traditional pair-wise meta-analysis (Table 2), we observed that chemotherapy had a lower CR and PR rate than nivolumab 3 mg/kg, pembrolizumab 10 mg/kg and pembrolizumab 2 mg/kg. CR, PR rate and ORR were also higher in patients under nivolumab 3 mg/kg treatment than chemotherapy (OR = 6.51, 95% CI: 1.95–21.76; OR = 2.57, 95% CI = 1.76–3.75; OR = 2.92, 95% CI: 2.02–4.21) and ipilimumab 3 mg/kg (OR = 3.99, 95% CI = 1.72 to 9.26; OR = 2.07, 95% CI = 1.449 to 2.97; OR = 2.29, 95% CI = 1.63 to 3.22). They were also higher for ipilimumab+nivolumab compared to ipilimumab 3 mg/kg. The disease was also found to be more stable under ipilimumab treatment than in nivolumab 3 mg/kg and ipilimumab+nivolumab treatment (OR = 0.49, 95% CI: 0.32–0.76; OR = 0.55, 95% CI: 0.37–0.80). Besides, the disease was more progressive under Ipilimumab 3 mg/kg (OR = 0.45, 95% CI = 0.33 to 0.60) or Nivolumab 3 mg/kg (OR = 0.60, 95% CI = 0.43 to 0.84) compared with that under Ipilimumab+Nivolumab. When comparing ORR, the application of ipilimumab+nivolumab was also observed to be more effective than the single use of nivolumab (OR = 1.32, 95% CI: 1.01–1.73). In regards to CR rate, tremelimumab 15 mg/kg was significantly more effective than tremelimumab 10 mg/kg (OR = 2.48, 95% CI: 1.64–3.76). However, other outcomes were not significantly approved.

**Table 2:** Meta-analysis results for pair-wise comparisons of clinical outcome

Comparisons	Complete Response	Partial Response	Stable Disease	Progressive Disease	Overall Response Rate
Tremelimumab 15 mg/kg vs. Chemotherapy	1.37 (0.54, 3.45)	1.04 (0.58, 1.85)	-	-	1.12 (0.68, 1.85)
Nivolumab 3 mg/kg vs. Chemotherapy	**6.51 (1.95, 21.76)**	**2.57 (1.76, 3.75)**	0.83 (0.59, 1.16)	0.85 (0.53, 1.38)	**2.92 (2.02, 4.21)**
Pembrolizumab 10 mg/kg vs. Chemotherapy	**6.18 (2.11, 18.12)**	**5.07 (2.31, 11.14)**	0.93 (0.55, 1.58)	0.77 (0.54, 1.09)	-
Pembrolizumab 2 mg/kg vs. Chemotherapy	**5.22 (1.76, 15.51)**	**4.23 (1.90, 9.38)**	0.96 (0.57, 1.64)	0.75 (0.53, 1.07)	-
Ipilimumab+Chemotherapy vs. Chemotherapy	2.34 (0.60, 9.14)	**1.65 (1.03, 2.65)**	0.98 (0.66, 1.46)	1.13 (0.46, 2.74)	-
Ipilimumab 10 mg/kg vs. Ipilimumab 3 mg/kg	1.62 (0.37, 7.08)	0.75 (0.31, 1.81)	0.62 (0.19, 2.01)	1.02 (0.56 1.87)	0.82 (0.23 2.98)
Nivolumab 3 mg/kg vs. Ipilimumab 3 mg/kg	**3.99 (1.72, 9.26)**	**2.07 (1.449, 2.97)**	**0.49 (0.32, 0.76)**	0.77 (0.58 1.02)	**2.29 (1.63, 3.22)**
Ipilimumab+Nivolumab vs. Ipilimumab 3 mg/kg	**5.52 (2.57, 11.87)**	**2.81 (2.01, 3.93)**	**0.55 (0.37, 0.80)**	**0.45 (0.33, 0.60)**	**3.29 (2.17, 5.00)**
Ipilimumab+Chemotherapy vs. Ipilimumab 3 mg/kg	1.70 (0.26, 11.07)	1.02 (0.54, 1.95)	1.23 (0.73, 2.10)	1.08 (0.73, 1.58)	1.21 (0.65, 2.26)
Ipilimumab+Chemotherapy vs. Ipilimumab 10 mg/kg	1.00 (0.06, 16.37)	0.65 (0.22, 1.96)	0.98 (0.39, 2.45)	1.15 (0.62, 2.13)	0.76 (0.27, 2.19)
Tremelimumab 15 mg/kg vs. Tremelimumab 10 mg/kg	**2.48 (1.64, 3.76)**	0.96 (0.18, 4.99)	1.12 (0.46, 2.68	0.96 (0.48, 1.91)	0.96 (0.22, 4.06)
Ipilimumab+Nivolumab vs. Nivolumab 3 mg/kg	1.29 (0.77, 2.17)	1.33 (0.99, 1.78)	1.21 (0.75, 1.96)	**0.60 (0.43, 0.84)**	**1.32 (1.01, 1.73)**
Pembrolizumab 2 mg/kg vs. Pembrolizumab 10 mg/kg	0.85 (0.46, 1.55)	0.88 (0.59, 1.33)	1.04 (0.68, 1.59)	0.94 (0.69, 1.28)	1.03 (0.53, 2.00)

**Figure 3:**
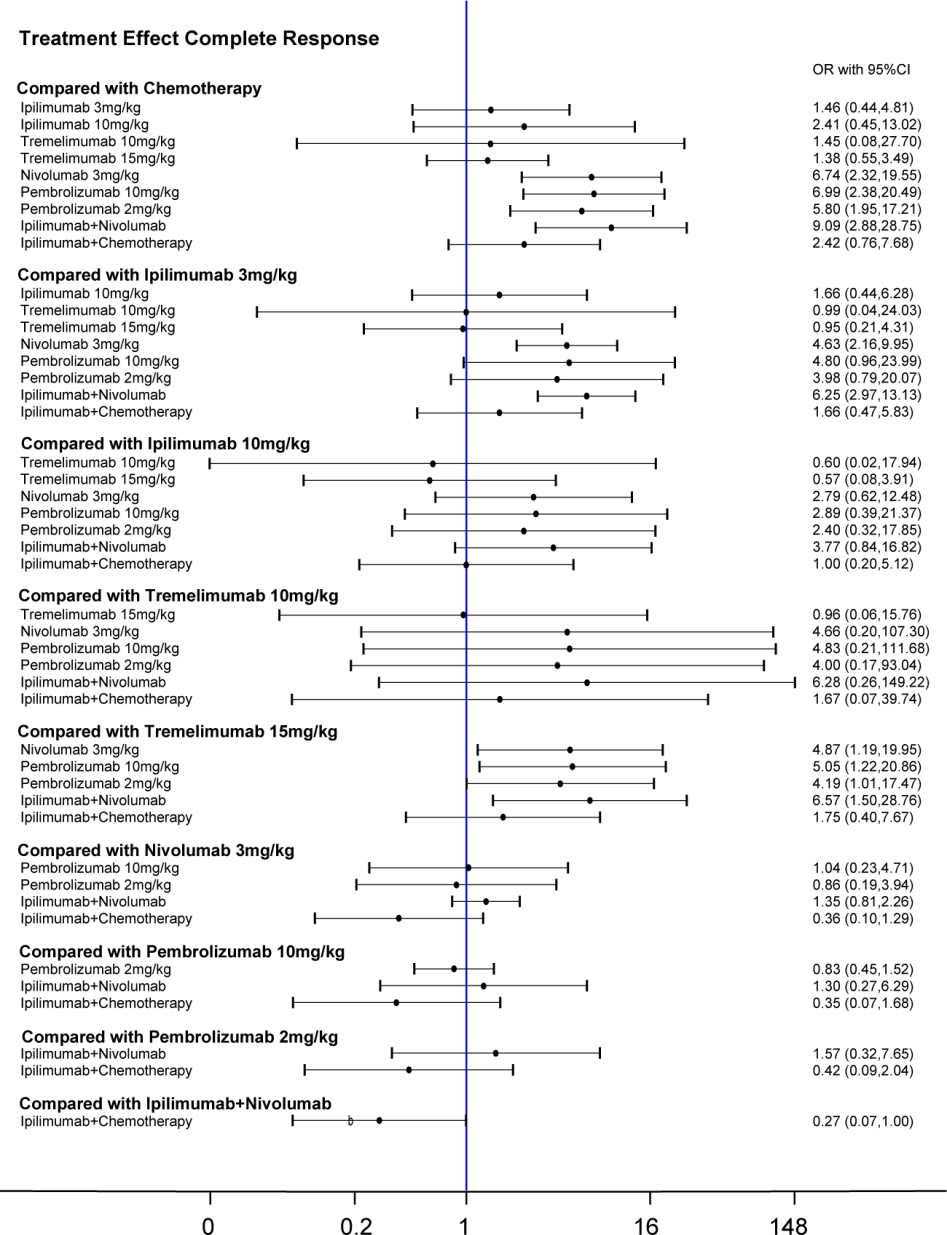
Forest plots for the correlations of the complete-response outcome of 10 interventions on melanoma

**Figure 4:**
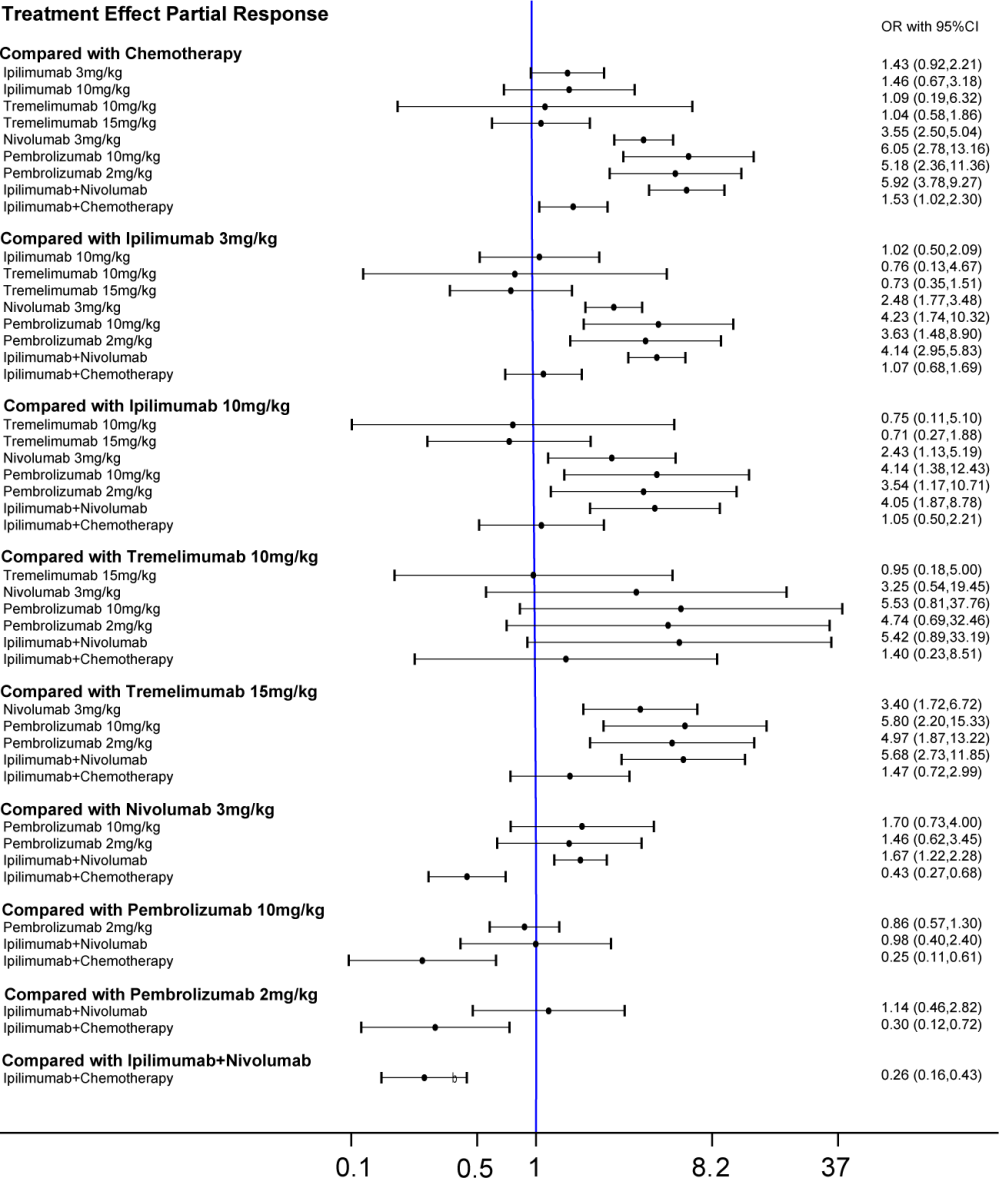
Forest plots for the correlations of the partial-response outcome of 10 interventions on melanoma

Our network meta-analysis combined both direct and indirect evidence into a single comparison to facilitate comparisons between interventions without a RCT. We observed that chemotherapy was less effective than nivolumab 3 mg/kg, pembrolizuma 10 mg/kg, pembrolizuma 2 mg/kg, ipilimumab+nivolumab as well as ipilimumab+ chemotherapy when comparing both CR and PR (Table 3). And 3 mg/kg, 10 mg/kg ipilimumab and ipilimumab + chemotherapy were found to have a lower PR than nivolumab 3 mg/kg, pembrolizuma 10 mg/kg, pembrolizuma 2 mg/kg and ipilimumab + nivolumab. Similar results were observed in patients under tremelimumab 15 mg/kg treatment, which has a lower CR and PR rate than the four therapies mentioned above. The single application of either ipilimumab or nivolumab had a lower CR than when combined (ipilimumab+ nivolumab). This result is consistent with that of pair-wise meta-analysis. Results from the network meta-analysis concerning clinical outcomes were illustrated in Figure 3 and Figure 4.

**Table 3:** Comparison of odds ratios of complete rate and partial rate for different interventions

**Chemotherapy**	1.46 (0.44, 4.81)	2.41 (0.45, 13.02)	1.45(0.08, 27.70)	1.38 (0.55, 3.49)	**6.74 (2.32, 19.55)**	**6.99 (2.38, 20.49)**	**5.80 (1.95, 17.21)**	**9.09 (2.88, 28.75)**	2.42 (0.76, 7.68)
1.43 (0.99, 2.21)	**Ipilimumab 3 mg/kg**	1.66 (0.44, 6.28)	0.99 (0.04, 24.03)	0.95 (0.21, 4.31)	**4.63 (2.16, 9.95)**	4.80 (0.96, 23.99)	3.98 (0.79, 20.07)	**6.25 (2.97, 13.13)**	1.66 (0.47. 5.83)
1.46 (0.92, 2.21)	1.02 (0.50, 2.09)	**Ipilimumab 10 mg/kg**	0.60 (0.02, 17.94)	0.57 (0.08,3.91)	**2.79 (0.62, 12.48)**	2.89 (0.39, 21.37)	2.40 (0.32, 17.85)	3.77 (0.84, 16.82)	1.00 (0.20, 5.12)
1.09 (0.19, 6.32)	0.76 (0.13, 4.67)	0.75 (0.11, 5.10)	**Tremelimumab 10 mg/kg**	0.96 (0.06, 15.76)	4.66 (0.20, 107.30)	4.83 (0.21, 111.68)	4.00 (0.17, 93.04)	6.28 (0.26, 149.22)	1.67 (0.07, 39.74)
1.04 (0.58, 1.86)	0.73 (0.35, 1.51)	0.71 (0.27, 1.88)	0.95 (0.18, 5.00)	**Tremelimumab 15 mg/kg**	**4.87 (1.19, 19.95)**	**5.05 (1.22, 20.86)**	**4.19 (1.01, 17.47)**	**6.57 (1.50, 28.76)**	1.75 (0.40, 7.67)
**3.55 (2.50, 5.04)**	**2.48 (1.77, 3.48)**	**2.43 (1.13, 5.19)**	3.25 (0.54, 19.45)	**3.40 (1.72, 6.72)**	**Nivolumab 3 mg/kg**	1.04 (0.23,4.71)	0.86 (0.19, 3.94)	1.35 (0.81, 2.26)	0.36 (0.10, 1.29)
**6.05 (2.78, 13.13)**	**4.23 (1.74, 10.32)**	**4.14 (1.38, 12.43)**	5.53 (0.81, 37.76)	**5.80 (2.20, 15.33)**	**1.70 (0.73, 4.00)**	**Pembrolizuma 10 mg/kg**	0.83 (0.45, 1.52)	1.30 (0.27, 6.29)	0.35 (0.07, 1.68)
**5.18 (2.36, 11.36)**	**3.63 (1.48, 8.90)**	**3.54 (1.17, 10.71)**	4.74 (0.69, 32.46)	**4.97 (1.87, 13.22)**	**1.46 (0.62, 3.45)**	0.86 (0.57, 1.30)	**Pembrolizuma 2 mg/kg**	1.57 (0.32, 7.65)	0.42 (0.09, 2.04)
**5.92 (3.78, 9.27)**	**4.14 (2.95, 5.83)**	**4.05 (1.87, 8.78)**	5.42 (0.89, 33.19)	**5.68 (2.73, 11.85)**	**1.67 (1.22,2.28)**	0.98 (0.40, 2.40)	1.14 (0.46, 2.82)	**Ipilimumab+ Nivolumab**	0.27 (0.07, 1.00)
**1.53 (1.02, 2.30)**	1.07 (0.68, 1.69)	1.05 (0.50, 2.21)	1.40 (0.23, 8.51)	1.47 (0.72, 2.99)	**0.43 (0.27, 0.68)**	**0.25 (0.11, 0.61)**	**0.30 (0.12, 0.72)**	**0.26 (0.16, 0.43)**	**Ipilimumab+ Chemotherapy**

Note: Odds ratios in the blue zone complete is for complete response and in the white zone for partial response. The column treatment is compared with the row treatment in blue squares while it is opposite in the white squares. Numbers in parentheses indicate 95% credible intervals.

### Adverse effects

We deemed fatigue, pruritus, rash, diarrhea and nausea as five important conditions in measuring adverse effects. Using traditional meta-analysis we observed the following (Table 4): Patients under chemotherapy were more likely to demonstrate symptoms of adverse effects than patients under immunotherapy. The occurrence of fatigue, diarrhea and rash was higher in chemotherapy patients than ipilimumab 3 mg/kg patients (fatigue: OR = 1.32, 95% CI: 1.03- 1.70; diarrhea: OR = 1.63, 95% CI: 1.27–2.08; rash: OR = 2.32, 95% CI: 1.73–3.10). Ipilimumab 3 mg/kg has a lower possibility of pruritus and diarrhea than nivolumab 3 mg/kg and pembrolizumab 10 mg/kg (pruritus: OR = 0.53, 95% CI: 0.38–0.76, OR = 0.55, 95% CI: 0.36–0.8, respectively; diarrhea: OR = 0.58, 95% CI: 0.41–0.83, OR = 0.64, 95% CI: 0.41–0.98, respectively). The combined application of ipilimumab and nivolumab could significantly attenuate the symptoms of diarrhea in ipilimumab treatment (OR = 1.33; 95% CI: 1.01–1.75), as well as decreased nausea and rash in nivolumab treatment (OR = 1.99, 95% CI: 1.32–2.99; OR = 1.56, 95% CI: 1.14–2.15).

**Table 4:** Meta-analysis results for pair-wise comparisons of adverse events

Comparisons	All adverse events	Fatigue	Pruritus	Diarrhea	Nausea	Rash
Chemotherapy vs. Ipilimumab 3 mg/kg	1.08 (0.90, 1.30)	1.32 (1.03, 1.70)	**2.91 (2.15, 3.92)**	**1.63 (1.27, 2.08)**	**1.41 (1.03, 1.91)**	**2.32 (1.73, 3.10)**
Chemotherapy vs. Tremelimumab 15 mg/kg	1.06 (0.85, 1.33)	0.90 (0.66, 1.21)	**6.23 (3.60, 10.80)**	**2.95 (2.10, 4.15)**	**0.69 (0.52, 0.92)**	0.90 (0.66, 1.21)
Chemotherapy vs. Nivolumab 3 mg/kg	1.02 (0.83, 1.27)	1.11 (0.77, 1.59)	**4.86 (1.56, 15.11)**	0.99 (0.66, 1.49)	**0.36 (0.26, 0.51)**	**5.12 (2.09, 12.52)**
Chemotherapy vs. Pembrolizumab 10 mg/kg	0.95 (0.69, 1.30)	0.83 (0.54, 1.27)	**6.92 (2.87, 16.69)**	1.34 (0.65, 2.76)	**0.28 (0.15, 0.52)**	2.22 (0.94, 5.25)
Chemotherapy vs. Pembrolizumab 2 mg/kg	0.86 (0.63, 1.19)	0.64 (0.41, 1.00)	**6.13 (2.53, 14.89)**	1.06 (0.50, 2.27)	**0.15 (0.07, 0.33)**	**2.61 (1.13, 6.05)**
Chemotherapy vs. Ipilimumab+Chemotherapy	1.04 (0.81, 1.34)	-	**3.34 (2.01, 5.56)**	**2.04 (1.34, 3.10)**	-	**3.62 (2.05, 6.37)**
Ipilimumab 3 mg/kg vs. Ipilimumab 10 mg/kg	0.73 (0.40, 1.34)	1.24 (0.58, 2.66)	**0.13 (0.03, 0.60)**	0.71 (0.33, 1.53)	0.83 (0.35, 1.98)	**0.17 (0.05, 0.62)**
Ipilimumab 3 mg/kg vs. Nivolumab 3 mg/kg	1.01 (0.81, 1.26)	1.23 (0.89, 1.69)	**0.53 (0.38, 0.76)**	**0.58 (0.41, 0.83)**	0.82 (0.53, 1.27)	0.79 (0.57, 1.10)
Ipilimumab 3 mg/kg vs. Pembrolizumab 10 mg/kg	-	0.92 (0.61, 1.38)	**0.55 (0.36, 0.84)**	**0.64 (0.41, 0.98)**	-	-
Ipilimumab 3 mg/kg vs. Ipilimumab+Nivolumab	1.02 (0.83, 1.25)	1.20 (0.90, 1.61)	0.99 (0.74, 1.32)	**1.33 (1.01, 1.75)**	1.45 (0.96, 2.12)	1.29 (0.97, 1.71)
Ipilimumab 3 mg/kg vs. Ipilimumab+Chemotherapy	1.19 (0.60, 2.34)	1.83 (0.88, 3.82)	0.66 (0.28, 1.54)	1.03 (0.56, 1.89)	1.50 (0.61, 3.67)	0.91 (0.47, 1.76)
Ipilimumab 10 mg/kg vs. Ipilimumab+Chemotherapy	-	-	-	1.38 (0.41, 4.56)	-	-
Tremelimumab 10 mg/kg vs. Tremelimumab 15 mg/kg	1.00 (0.19, 5.36)	0.79 (0.31, 1.99)	1.18 (0.42, 3.35)	1.11 (0.54, 2.30)	0.89 (0.37, 2.15)	0.97 (0.45, 2.07)
Tremelimumab 15 mg/kg vs. Ipilimumab+Nivolumab	-	-	**1.77 (1.24, 2.53)**	**2.32 (1.65, 3.25)**	-	-
Nivolumab 3 mg/kg vs. Ipilimumab+Nivolumab	1.01 (0.81, 1.26)	1.03 (0.76, 1.41)	-	-	**1.99 (1.32, 2.99)**	**1.56 (1.14, 2.15)**
Pembrolizumab 10 mg/kg vs. Pembrolizumab 2 mg/kg	0.91 (0.66, 1.26)	1.39 (0.26, 7.40)	0.88 (0.54, 1.44)	0.8 (0.44, 1.59)	0.54 (0.22, 1.29)	1.16 (0.61, 2.21)

**Figure 5:**
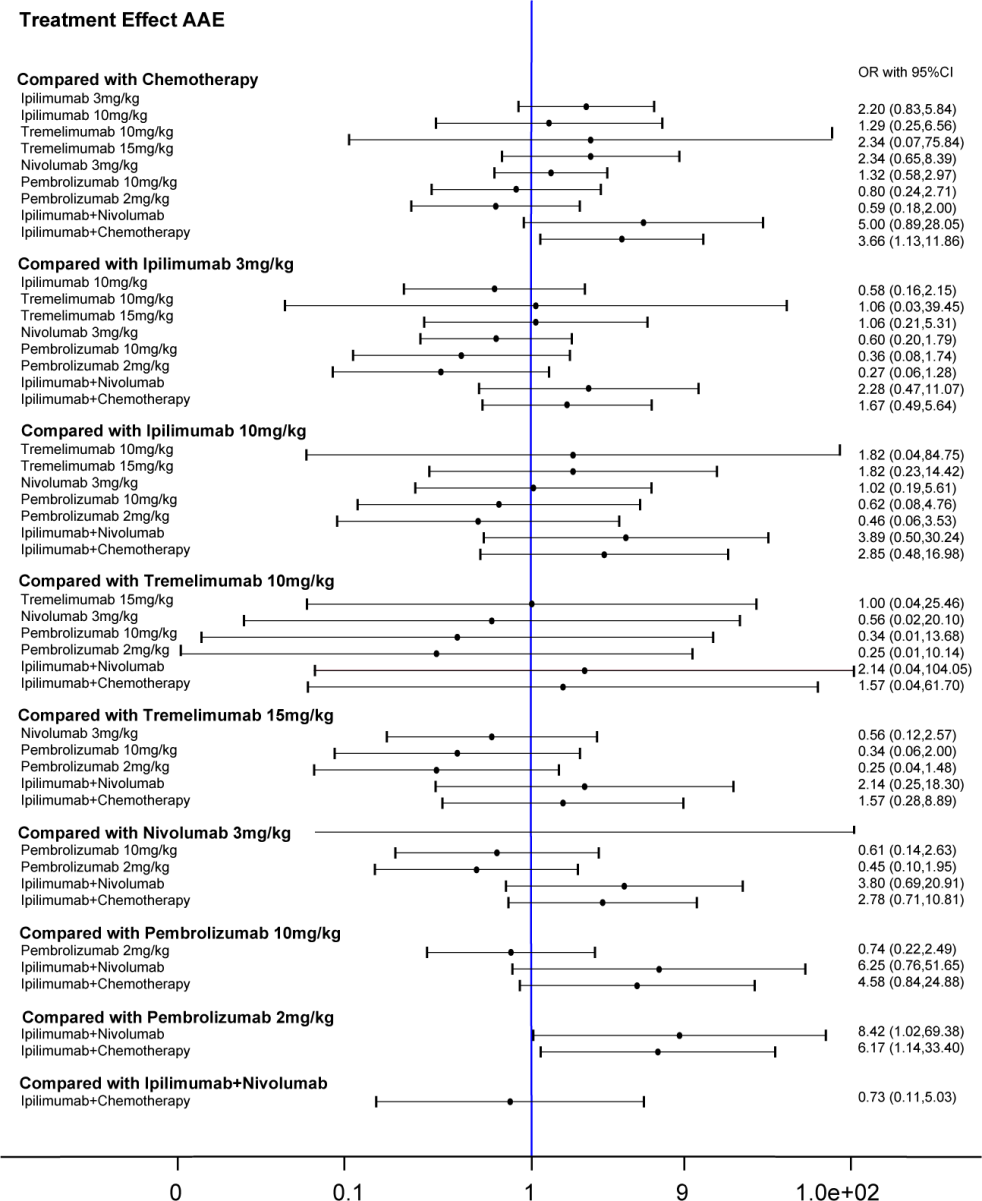
Forest plots for the correlations of the all-adverse-event outcome of 10 interventions on melanoma

**Figure 6:**
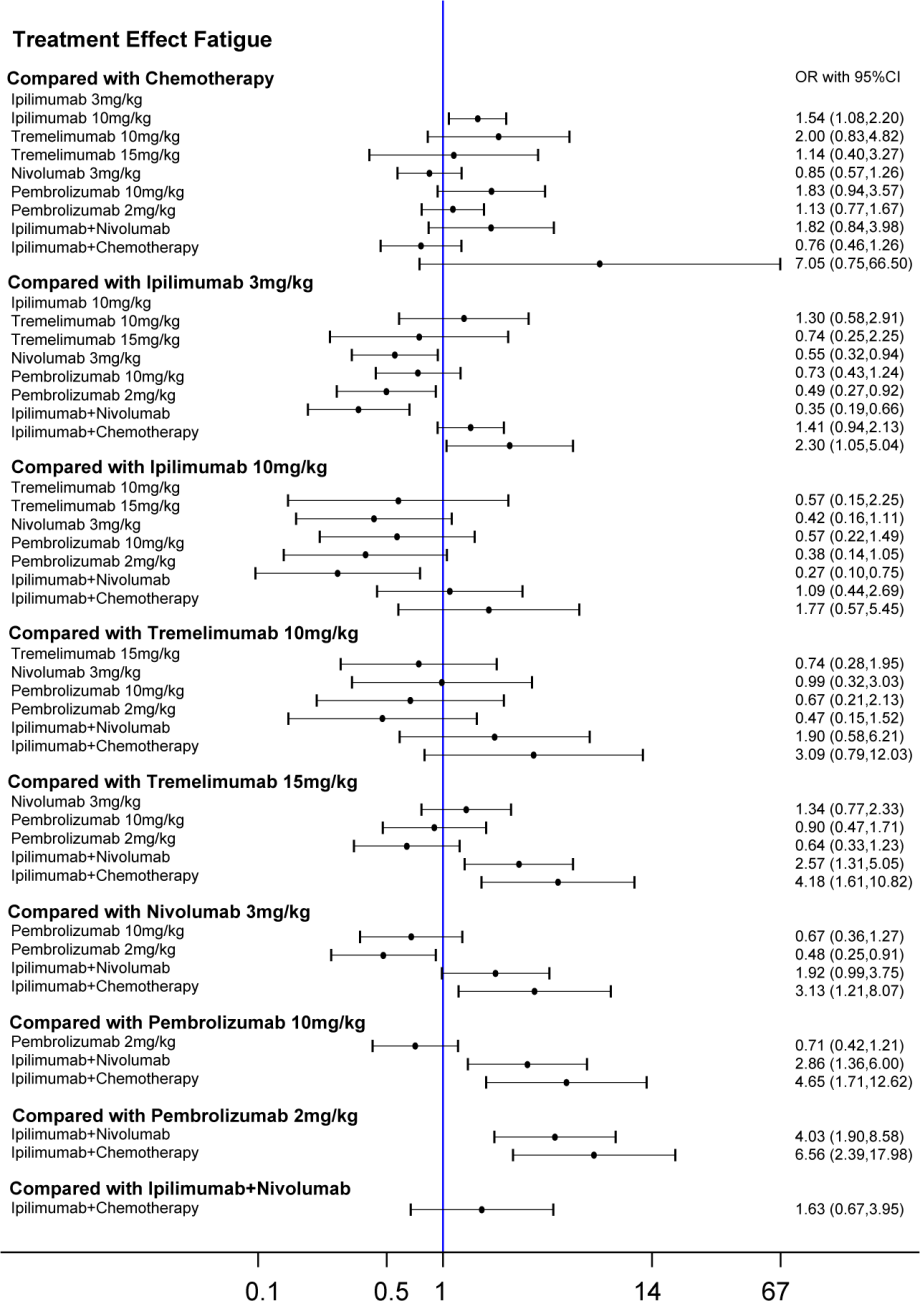
Forest plots for the correlations of the fatigue outcome of 10 interventions on melanoma

Results from the network meta-analysis were illustrated in Figure 5 and Figure 6. Taking fatigue into account, ipilimumab 3 mg/kg and ipilimumab+ nivolumab triggered more fatigues than pembrolizumab 10 mg/kg, 2 mg/kg and tremelimumab 15 mg/kg (Table 5). Patients under ipilimumab+ chemotherapy were more likely to have fatigue compared to tremelimumab 15 mg/ kg, nivolumab 3 mg/kg, pembrolizumab 10 mg/kg, pembrolizumab 2 mg/kg and ipilimumab 3 mg/kg. For all advert events, we found that pembrolizumab 2 mg/kg was less likely to cause advert events than ipilimumab+ chemotherapy and ipilimumab + nivolumab. With respect to rash, Ipilimumab+ nivolumab resulted in a significantly higher risk of rash than chemotherapy, ipilimumab 3 mg/kg, ipilimumab 10 mg/kg, tremelimumab 10 mg/ kg, tremelimumab 15 mg/kg and nivolumab 3 mg/kg (Table 6). And Ipilimumab 3 mg/kg, nivolumab 3 mg/kg, and ipilimumab+ chemotherapy treatment resulted in higher possibilities of rash than ipilimumab 10 mg/kg, tremelimumab 10 mg/kg and 15 mg/kg. In addition, tremelimumab 15 mg/kg had a significant higher possibility of diarrhea than all therapies except Tremelimumab 10 mg/kg and Ipilimumab+ Nivolumab (Table 6).

**Table 5:** Comparison of odds ratios of adverse events and fatigue for different interventions

**Chemotherapy**	2.20 (0.83, 5.84)	1.29 (0.25, 6.56)	2.34 (0.07, 75.84)	2.34 (0.65, 8.39)	1.32 (0.58, 2.97)	0.80 (0.24, 2.71)	0.59 (0.18, 2.00)	5.00 (0.89, 28.05)	**3.66 (1.13, 11.86)**
1.54 (1.08, 2.20)	**Ipilimumab 3 mg/kg**	0.58 (0.16, 2.15)	1.06 (0.03, 39.45)	1.06 (0.21, 5.31)	0.60 (0.20, 1.79)	0.36 (0.08, 1.74)	0.27 (0.06, 1.28)	2.28 (0.47, 11.07)	**1.67 (0.49, 5.64)**
2.00 (0.83, 4.82)	1.30 (0.58, 2.91)	**Ipilimumab 10 mg/kg**	1.82 (0.04, 84.75)	1.82 (0.23, 14.42)	1.02 (0.19, 5.61)	0.62 (0.08, 4.76)	0.46 (0.06, 3.53)	3.89 (0.50, 30.24)	**2.85 (0.48, 16.98)**
1.14 (0.40, 3.27)	0.74 (0.25, 2.25)	0.57 (0.15, 2.25)	**Tremelimumab 10 mg/kg**	1.00 (0.04, 25.46)	0.56 (0.02, 20.10)	0.34 (0.01, 13.68)	0.25 (0.01, 10.14)	2.14 (0.04, 104.05)	**1.57 (0.04, 61.70)**
0.85 (0.57, 1.26)	**0.55 (0.32, 0.94)**	0.42 (0.16, 1.11)	0.74 (0.26, 1.95)	**Tremelimumab 15 mg/kg**	0.56 (0.12, 2.57)	0.34 (0.06. 2.00)	0.25 (0.04, 1.48)	2.14 (0.25, 18.30)	**1.57 (0.28, 8.89)**
1.83 (0.94, 3.57)	**0.73 (0.43, 1.24)**	0.57 (0.22, 1.49)	0.99 (0.32, 3.03)	1.34 (0.77, 1.27)	**Nivolumab 3 mg/kg**	0.61 (0.14, 2.63)	0.45 (0.10, 1.95)	3.80 (0.69, 20.91)	**2.78 (0.71, 10.81)**
1.13 (0.77, 1.67)	**0.49 (0.27, 0.92)**	0.38 (0.14, 1.05)	0.67 (0.21, 2.13)	0.90 (0.47, 1.71)	0.67 (0.36, 1.27)	**Pembrolizuma 10 mg/kg**	0.74 (0.22, 2.49)	6.25 (0.76, 51.65)	**4.58 (0.84, 24.88)**
1.82 (0.84, 3.96)	**0.35 (0.19, 0.66)**	**0.27 (0.10, 0.75)**	0.47 (0.15, 1.52)	0.64 (0.33, 1.23)	**0.48 (0.25, 0.91)**	0.71 (0.42, 1.21)	**Pembrolizuma 2 mg/kg**	**8.42 (1.02, 69.38)**	**6.17 (1.14, 33.40)**
0.76 (0.46, 1.26)	1.41 (0.94, 2.12)	1.09 (0.44, 2.69)	1.90 (0.58, 6.21)	**2.57 (1.31, 5.05)**	1.92 (0.99, 3.75)	**2.86 (1.36, 6.00)**	**4.03 (1.90, 8.58)**	**Ipilimumab+ Nivolumab**	0.73 (0.11, 5.03)
7.05 (0.75, 66.50)	**2.30 (1.05, 5.04)**	1.77 (0.57, 5.45)	3.09 (0.79, 12.03)	**4.18 (1.61, 10.82)**	**3.13 (1.21, 8.07)**	**4.65 (1.71, 12.62)**	**6.56 (2.39, 17.89)**	**1.63 (0.67, 3.95)**	**Ipilimumab+ Chemotherapy**

Note: Odds ratios in the blue zone is for all adverse events and in the white zone for fatigue. The column treatment is compared with the row treatment in blue squares while it is opposite in the white squares. Numbers in parentheses indicate 95% credible intervals.

**Table 6:** Comparison of odds ratios of diarrhea and rash for different interventions

**Chemotherapy**	**2.23 (1.79, 2.78)**	1.53 (0.77, 3.03)	**4.12 (1.71, 9.91)**	**4.96 (3.46, 7.11)**	1.04 (0.77, 1.40)	1.31 (0.87, 1.97)	1.05 (0.54, 2.04)	**3.56 (2.53, 5.02)**	**2.44 (1.72, 3.47)**
**3.91 (2.42, 6.32)**	**Ipilimumab 3 mg/kg**	0.68 (0.35, 1.33)	1.85 (0.75, 4.56)	**2.22 (1.46, 3.39)**	**0.47 (0.35, 0.62)**	**0.59 (0.40, 0.86)**	**0.47 (0.24, 0.92**)	**1.60 (1.20, 2.13)**	1.09 (0.76, 1.58)
0.54 (0.13, 2.28)	**0.14 (0.04, 0.54)**	**Ipilimumab 10 mg/kg**	2.70 (0.89, 8.23)	**3.25 (1.50, 7.05)**	0.68 (0.33, 1.40)	0.86 (0.40, 1.84)	0.69 (0.27, 1.76)	**2.33 (1.13, 4.81)**	1.60 (0.79, 3.24)
0.91 (0.32, 2.63)	**0.23 (0.07, 0.74)**	1.68 (0.28, 9.93)	**Tremelimumab 10 mg/kg**	1.20 (0.54, 2.68)	**0.25 (0.10, 0.64)**	**0.32 (0.12, 0.84)**	**0.25 (0.08, 0.77)**	0.87 (0.34, 2.22)	0.59 (0.23, 1.53)
0.85 (0.49, 1.47)	**0.22 (0.10, 0.45)**	1.56 (0.34, 7.25)	0.93 (0.38, 2.30)	**Tremelimumab 15 mg/kg**	**0.21 (0.13, 0.33)**	**0.26 (0.15, 0.45)**	**0.21 (0.10, 0.45)**	0.72 (0.44, 1.18)	**0.49 (0.30, 0.81)**
**3.52 (1.76, 7.03)**	0.90 (0.53, 1.51)	**6.49 (1.52, 27.65)**	**3.87 (1.11, 13.49)**	**4.16 (1.72, 10.08)**	**Nivolumab 3 mg/kg**	1.26 (0.79, 2.00)	1.01 (0.49, 2.06)	**3.43 (2.47, 4.75)**	**2.35 (1.51, 3.65)**
2.38 (0.91, 6.23)	0.61 (0.21, 1.78)	4.39 (0.78, 24.68)	2.62 (0.63, 10.94)	2.82 (0.93, 8.53)	0.68 (0.21, 2.21)	**Pembrolizuma 10 mg/kg**	0.80 (0.42, 1.52)	**2.72 (1.70, 4.37)**	1.87 (1.12, 3.12)
**2.80 (1.09, 7.21)**	0.71 (0.25, 2.07)	5.15 (0.92, 28.80)	3.07 (0.74, 12.73)	**3.31 (1.11, 9.89)**	0.79 (0.25, 2.57)	1.17 (0.54, 2.54)	**Pembrolizuma 2 mg/kg**	**3.40 (1.65, 7.03)**	**2.33 (1.11, 4.91)**
**6.71 (3.27, 13.75)**	**1.71 (1.04, 2.83)**	**12.36(2.92, 52.30)**	7.36 (2.08, 26.07)	**7.93 (3.21, 19.60)**	**1.90 (1.13, 3.21)**	2.81 (0.85, 9.32)	2.40 (0.73, 7.89)	**Ipilimumab+ Nivolumab**	0.69 (0.43, 1.08)
**3.94 (2.24, 6.91)**	1.01 (0.56, 1.80)	**7.25 (1.66, 31.66)**	4.32 (1.30, 14.32)	**4.66 (2.12, 10.23)**	1.12 (0.51, 2.46)	1.65 (0.54, 5.03)	1.41 (0.47, 4.24)	0.59 (0.27, 1.30)	**Ipilimumab + Chemotherapy**

Note: Odds ratios in the blue zone complete is for diarrhea and in the white zone for rash. The column treatment is compared with the row treatment in blue squares while it is opposite in the while squares. Numbers in parentheses indicate 95% credible intervals.

### Cumulative analysis and publication bias

**Figure 7:**
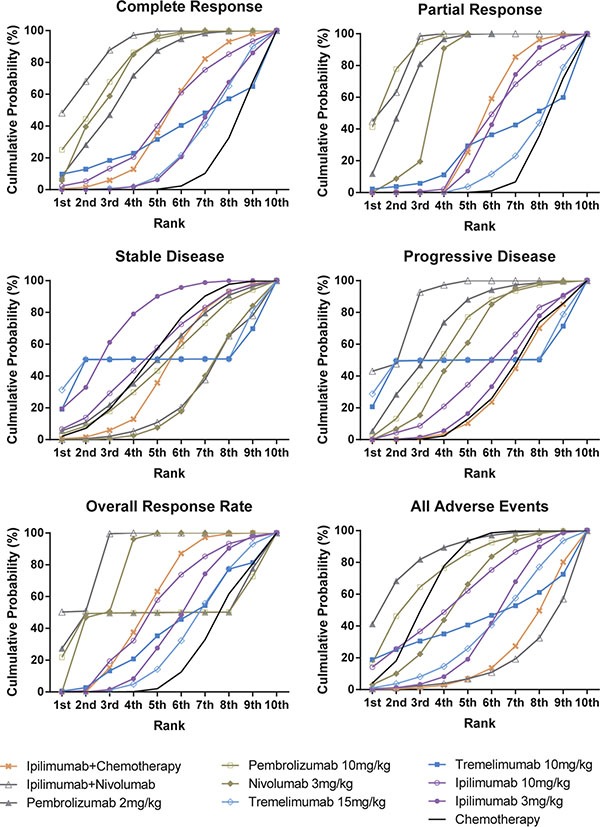
Rankograms showing cumulative probability of each strategy having each specific rank (1–10) for clinical response Ranking indicates the probability to be the best treatment, the second best, the third best and so on. Rank 1st is best and Rank 10th is worst.

**Figure 8:**
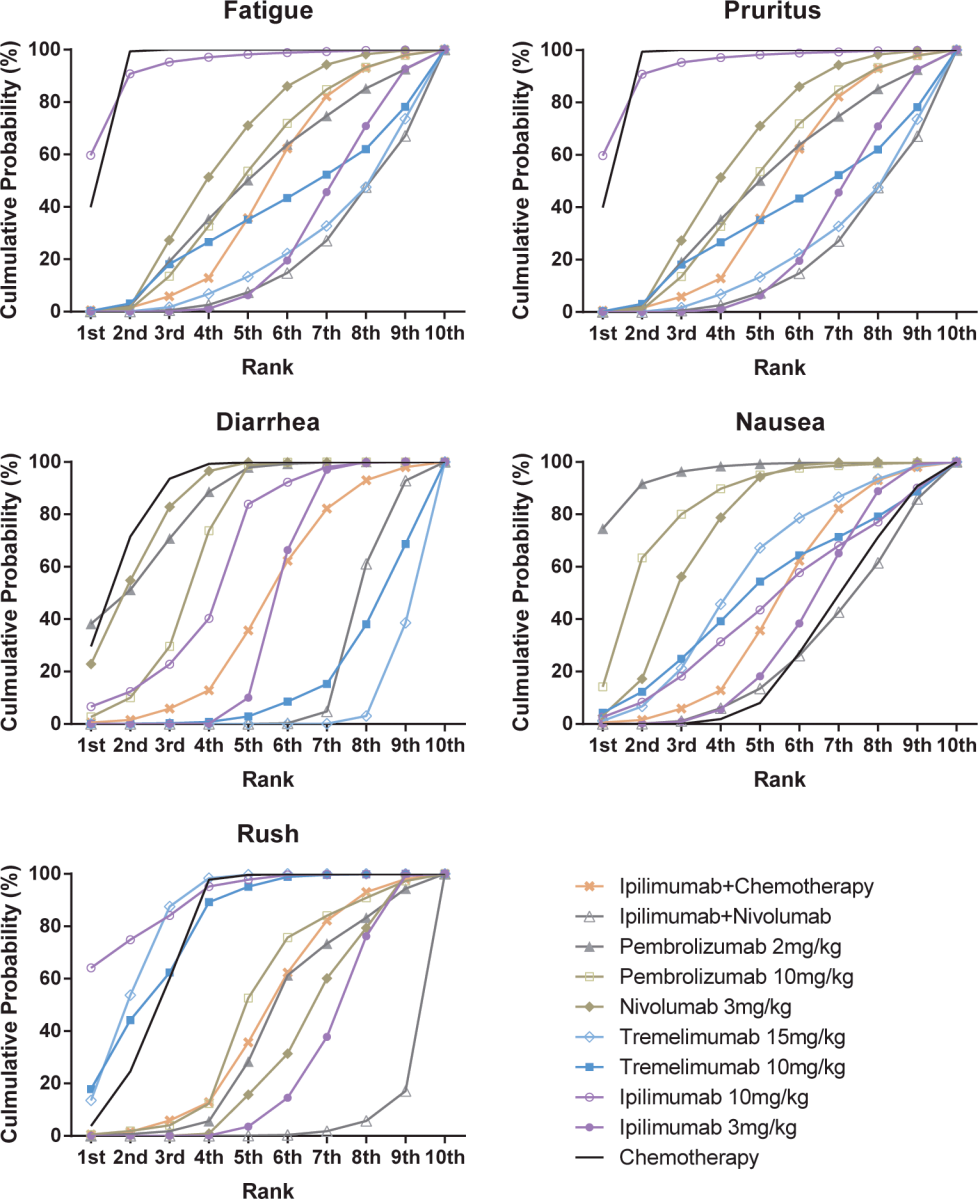
Rankograms showing cumulative probability of each strategy having each specific rank (1–10) for adverse events Ranking indicates the probability to be the best treatment, the second best, the third best and so on. Rank 1st is best and Rank 10th is worst.

We generated the SUCRA curve to calculate and rank the cumulative probability of all treatments and outcomes. The results are presented in Table 7, Figure 7 and Figure 8. We observed that chemotherapy ranked the lowest in regard to CR, PR and ORR. Besides, it also ranked the second lowest in progressive disease, only prior to ipilimumab+chemotherapy. Tremelimumab 15 mg/kg also ranked low in CR, PR and ORR, but it had a relatively high rank in progressive disease. Although ipilimumab+nivolumab had the highest response rate, patients using ipilimumab+nivolumab were not easily to show stable disease qualities.

**Table 7:** NMA results of SUCRA for all intervention outcomes of melanoma

Treatment	CR	PR	SD	PD	ORR	AAE	Fatigue	Pruritus	Rash	Diarrhea	Nausea
Chemotherapy	12.7	12.7	53.8	27.6	21.4	71.2	62.4	93.2	76.2	88.3	27.6
Ipilimumab 3 mg/kg	25.1	35.8	76.9	31.3	38.9	36.7	34.8	26.2	25.7	41.5	35.1
Ipilimumab 10 mg/kg	43.5	35.5	55.0	39.8	51.0	60.0	24.1	93.2	90.6	61.8	44.1
Tremelimumab 10 mg/kg	33.8	26.8	49.4	49.1	36.3	42.5	52.8	35.5	78.5	15.0	48.7
Tremelimumab 15 mg/kg	25.4	18.3	52.3	50.9	31.0	35.8	75.3	22.0	83.6	4.6	55.4
Nivolumab 3 mg/kg	75.8	68.8	22.9	56.0	77.1	57.9	53.1	58.9	31.9	84.1	72.0
Pembrolizumab 10 mg/kg	80.1	90.4	46.1	61.9	49.2	75.2	79.8	49.8	46.6	68.3	82.0
Pembrolizumab 2 mg/kg	71.2	81.7	50.9	70.3	50.4	85.5	96.5	47.1	38.9	82.9	95.6
Ipilimumab + Nivolumab	88.9	89.5	23.2	86.7	89.0	15.0	16.8	18.6	2.7	17.7	26.4
Ipilimumab + Chemotherapy	43.5	40.6	69.5	26.5	55.7	20.2	4.4	55.5	25.2	36.0	13.2

Outcomes: CR complete response; PR partial response; SD stable disease; PD progressive disease; ORR overall response rate; AAE all adverse events.

Patients under pembrolizumab 2 mg/kg treatment showed the lowest probability of suffering adverse effects, especially fatigue, diarrhea and nausea. The combination of Ipilimumab and nivolumab was found to be most dangerous as it had a high rank in all five of the adverse effects, they amplified the effects of pruritus, diarrhea and nausea compared to single use of nivolumab. Application of Ipilimumab 10 mg/kg was closely related to trigger fatigue, and chemotherapy was closely related to trigger nausea. Therefore, combined application of ipilimumab and chemotherapy caused a high possibility of both fatigue and nausea.

**Figure 9:**
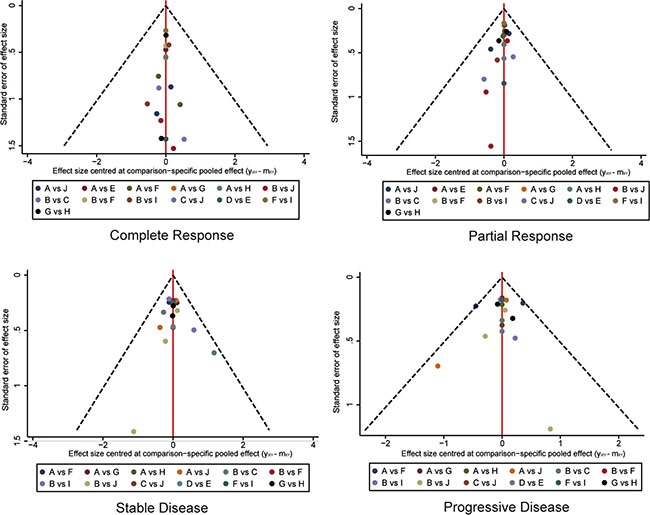
Funnel plot for assessing publications bias of clinical response A Chemotherapy; B Ipilimumab 3 mg/kg; C Ipilimumab 10 mg/kg; D Tremelimumab 10 mg/kg; E Tremelimumab 15 mg/kg; F Nivolumab 3 mg/kg; G Pembrolizumab 10 mg/kg; H Pembrolizumab 2 mg/kg; I Ipilimumab+Nivolumab; J Ipilimumab+Chemotherapy.

**Figure 10:**
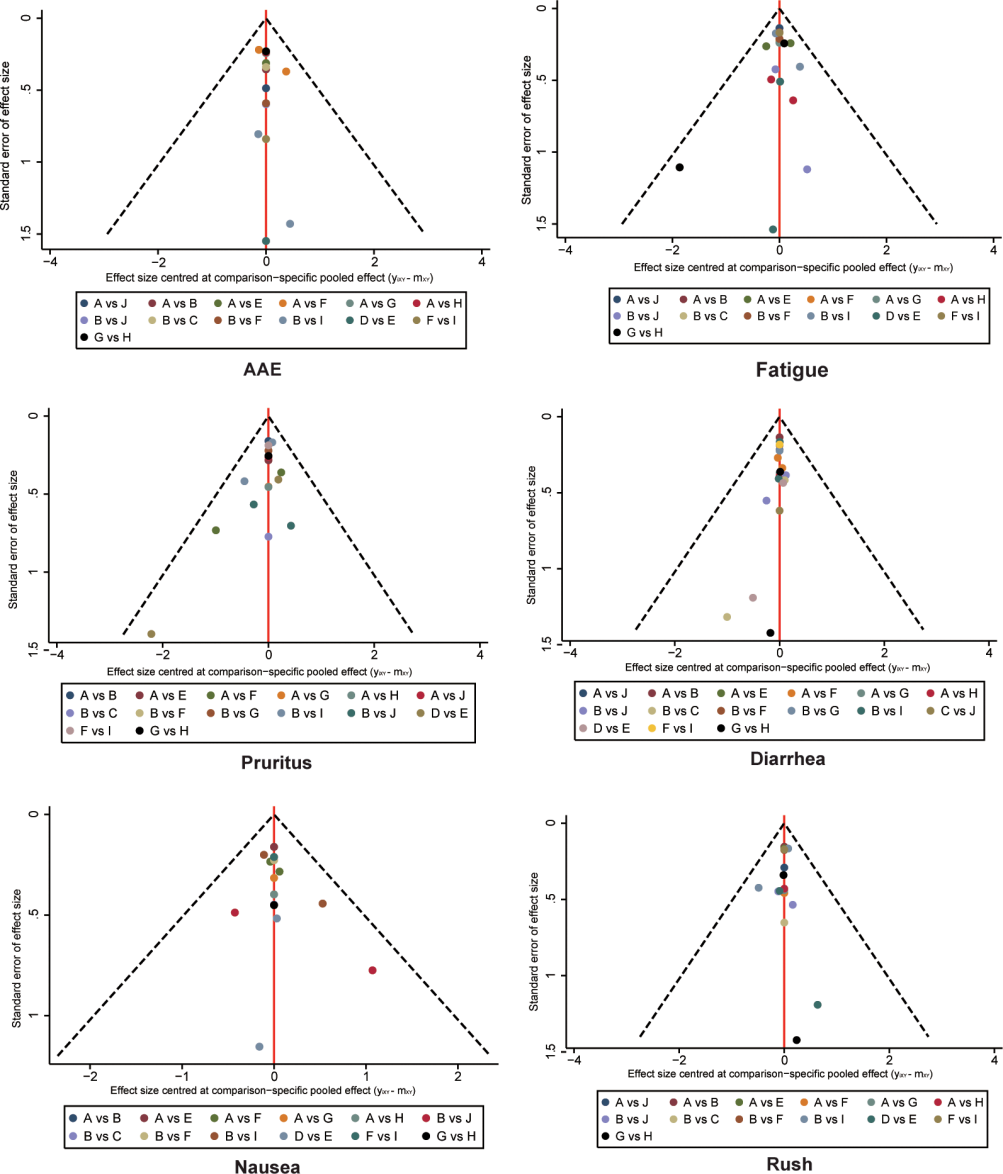
Funnel plot for assessing publications bias of adverse events A Chemotherapy; B Ipilimumab 3 mg/kg; C Ipilimumab 10 mg/kg; D Tremelimumab 10 mg/kg; E Tremelimumab 15 mg/kg; F Nivolumab 3 mg/kg; G Pembrolizumab 10 mg/kg; H Pembrolizumab 2 mg/kg; I Ipilimumab+Nivolumab; J Ipilimumab+Chemotherapy.

Funnel plots for publication bias were illustrated in Figure 9 and Figure 10. No significant bias was observed in publication.

## DISCUSSION

In the current study, we investigated the therapeutic value of immunotherapy and chemotherapy on melanoma. Interventions were categorized as chemotherapy, ipilimumab 3 mg/kg, ipilimumab 10 mg/kg, tremelimumab 10 mg/kg, tremelimumab 15 mg/kg, nivolumab 3 mg/kg, pembrolizumab 10 mg/kg, pembrolizumab 2 mg/kg, ipilimumab + nivolumab and ipilimumab + chemotherapy. The outcomes assessed included CR, PR, SD, PD, ORR and adverse effects including fatigue, pruritus, rash, diarrhea and nausea. A total of 20 RCTs and 6,442 cases were involved in the study.

Chemotherapy agents such as temozolomide, dacarbazine, high-dose IL-2, paclitaxel and carboplatin are commonly used in the treatment of melanoma. According to the results, we observed that chemotherapy had the lowest response rate and was also closely related to PD than immunotherapy. Furthermore, chemotherapy had a high rank in adverse effects, especially nausea. Considering its relation to the progressive disease category and strong adverse effects, chemotherapy may not be an ideal treatment for patients with melanoma.

Tremelimumab and ipilimumab are human monoclonal antibodies of CTLA4. CTLA4 is a member of the immunoglobulin superfamily that encodes protein transmitting inhibitory signals to T cells. Monoclonal antibodies targeting CTLA4 can increase T cell function and induce tumor regression [33]. In a phase I/II study, both tremelimumab and ipilimumab illustrated a high therapeutic value in melanoma treatment [34, 35]. However, in a phase III trial, severe life threatening adverse effects were observed in patients dosed with ipilimumab. This observation prevented its application in our current study [36]. Nivolumab and pembrolizumab are antibodies of PD-1. PD-1 is expressed on activated T lymphocytes, B lymphocytes and NK cells and works as an immune checkpoint inhibitor. It acts by binding to its two ligands PD-L1 and PD-L2 to induce T cell tolerance [37]. Nivolumab and pembrolizumab have good performance in early phase studies and have been approved for the use in therapy of melanoma [38, 39]. In our results, tremelimumab 15 mg/kg was observed to have a low response rate, a low progressive diseases rank and a high incidence rate of adverse effects, with the exception of fatigue and rash. Our results also indicated that the combined application of ipilimumab and nivolumab had the highest response rate among all studied therapies; however, it was considered as the most dangerous form of therapy for its high rank in all adverse effects, as well as the second lowest stable disease rank. We strongly recommend the use of pembrolizumab, a drug that is used in immunotherapy. A low dose of pembrolizumab demonstrated a high response rate and had the lowest possibility of adverse effects, particularly fatigue, diarrhea and nausea. Moreover, it ranked the second highest in the progressive disease category.

We observed that when ipilimumab and nivolumab were applied simultaneously, both the response rate and adverse effects were amplified. This result was also supported by pre-clinical studies [40]. It was also observed that nivolumab had better outcomes in patients that had never received treatment before; whereas ipilimumab + nivolumab were more effective on patients with PD-L1-negative malignancies [26]. Furthermore, adverse effects were also magnified in patients using both ipilimumab and chemotherapy. Increased toxicity may limit the use of combined therapy.

There are also some limitations of our meta-analysis that should be noted. Although this is a large-scale meta-analysis concerning the therapeutic value of immunotherapy and chemotherapy on melanoma, the samples size is quite limited. Subgroup analysis based on the ethnicity and age of patients was not performed. The lack of standardized agents in chemotherapy may also affect the reliability and validity of our results. Meanwhile, there are no sufficient data on SD and PD, which leads to several low reliable results. For example ipilimumab + nivolumab ranked low in SD but highest in PD among all. Further test of SD and PD should be determined to optimize the result. Furthermore, our study only compared the therapeutic value of CTLA4 and PD-1 inhibitors. The MEK inhibitor trametinib and BRAF inhibitors, such as vemurafenib and dabrafenib were not enrolled in the comparison as we failed to retrieve related RCTs.

In conclusion, our network meta-analysis results indicate that the combined use of immunotherapy and pembrolizumab is the treatment of choice. This is due to its high efficacy rate and minimal adverse effects. The combined application of ipilimumab and nivolumab may generate a higher incidence rate of adverse effects. Since the combined application of ipilimumab and nivolumab had the highest response rate, it should be applied to patients that do not respond to other treatments. Chemotherapy had a low response rate, high adverse effects and progressive diseases qualities and therefore, it is not a preferred treatment for patients with melanoma.

## MATERIALS AND METHODS

### Search strategy and inclusion criteria

The Cochrane library, PubMed and Embase databases were used to search for any relevant articles containing the key terms: melanoma, chemotherapy, immunotherapy, ipilimumab, tremelimumab, nivolumab, pidilizumab, pembrolizumab, and randomized controlled trial. Retrieved articles were predominately screened by two independent researchers (Dr. Xinhua Wang and Dr. Ziwen Long) based on titles and abstracts. We also manually reviewed the reference list for related studies to avoid improper exclusion.

Articles were deemed relevant to the current study if they met the following criteria: I) experiments were randomized controlled trails (RCTs); II) all cases were above the age of 18; III) treatment including medication and dosage was clearly described; IV) the diagnosis of melanoma is pathologically confirmed and staged according to AJCC guidelines [9]; V) outcomes including complete response (CR), partial response (PR), stable disease (SD), progressive disease (PD), overall response rate (ORR) and all adverse effects (AAE) were evaluated.

The quality of all enrolled studies was assessed for bias risk using the Cochrane Collaboration's tool [41]. Studies were evaluated on their design and completeness, which included sequence generation, selective reporting, incomplete outcome data, allocation concealment, blinding and other sources of bias. Only studies with low bias risk were used in our study.

### Data extraction

Two researchers (Dr. Xinhua Wang and Dr. Ziwen Long) independently extracted relevant data from the qualified articles. The data included the name of the first author, year of publication, trial ID, melanoma phase, medication used, dosage and the clinical outcome. All the data were documented for further analysis. In this study, CR, PR, SD, PD and ORR were considered as outcomes related to the effectiveness of therapy. Fatigue, pruritus, rash, diarrhea and nausea were considered as major adverse effects. A third researcher was also involved if any discrepancies arose.

### Statistical analysis

Firstly, we performed a traditional pair-wise meta-analysis to evaluate therapeutic value of each treatment. Odds ratios (ORs) and corresponding 95% confidence intervals (CIs) were calculated. The heterogeneity was determined by using Cochran's *Q*-statistic or *I*^2^ test and a *P* < 0.05 or *I*^2^ > 50% indicated the existence of heterogeneity. A fixed-effects model (*Mantel-Haenszel* method) was used for studies without significant heterogeneity, and a random-effects model (*Der Simonian-Laird* method) was applied to studies with significant heterogeneity.

Subsequently, a Bayesian network meta-analysis was performed to combine both direct and indirect evidence into a single comparison, using *Markov chain Monte Carlo* methods. Network plots were built to illustrate the comparison of various drugs. The results were illustrated by cumulative ORs and corresponding 95% credible intervals (CrIs). The probabilities and outcomes of each treatment were ranked and summarized using the surface under the cumulative ranking curve (SUCRA) as previously described [42].

Publication bias of involved articles was assessed using the funnel plot and Egger's test. The existence of publication bias was indicated by a *P* < 0.05. In traditional pair-wise meta-analysis, we used the STATA version 12.0 (Stata Corp, College Station, TX, USA) software. And WinBUGS (MRC Bio-statistics Unit, Cambridge, UK) software was used for calculations during network meta-analysis.

## SUPPLEMENTARY MATERIALS


