# Supplementary material for: The evaluation of immunotherapy and chemotherapy treatment on melanoma: a network meta-analysis
Source: Oncotarget. 2016 Nov 10;7(49):81493–511. doi: 10.18632/oncotarget.13277 (PMC5348408; doi:10.18632/oncotarget.13277)
Supplement: Supplementary file 1 [file oncotarget-07-81493-s001.pdf]

The evaluation of immunotherapy and chemotherapy treatment on melanoma: a network meta-analysis

Supplementary Materials

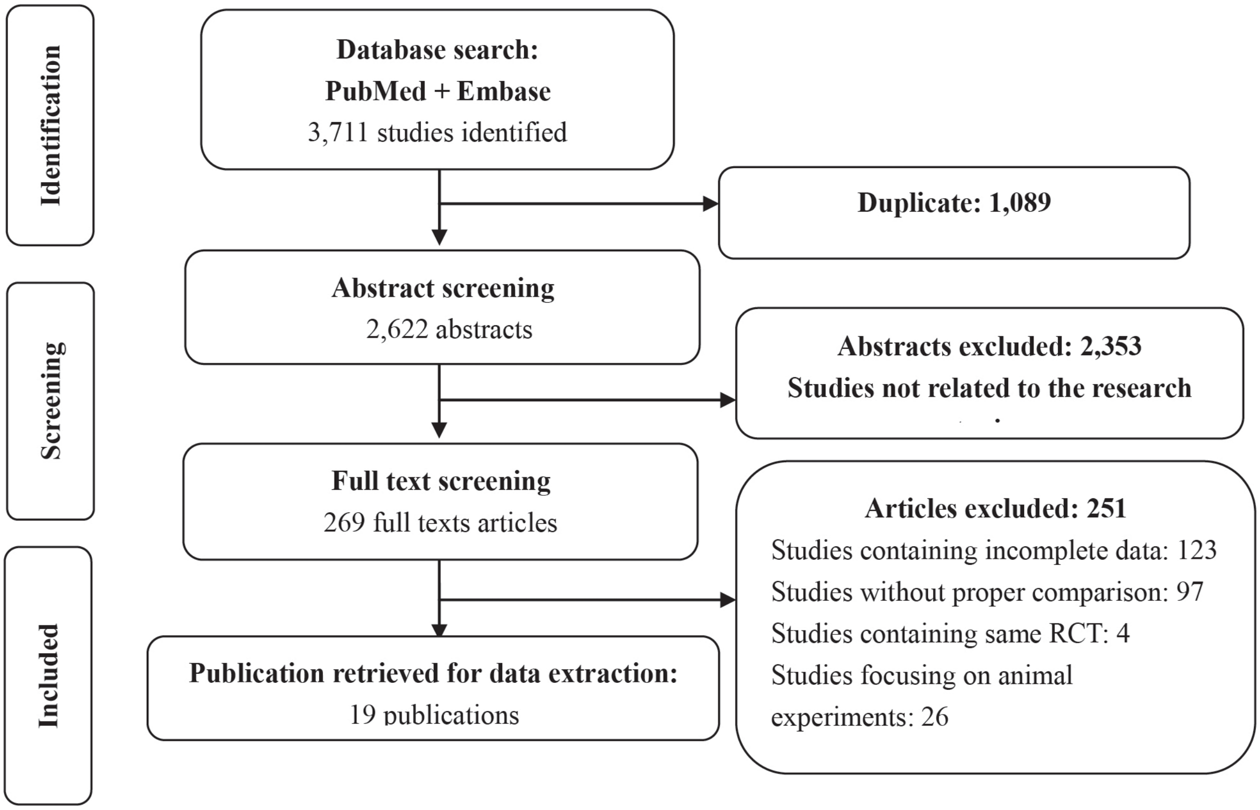

Supplementary Figure S1: Flow chart depicting the selection process.
